# Molecular Characteristics and Expression Patterns of Carotenoid Cleavage Oxygenase Family Genes in Rice (*Oryza sativa* L.)

**DOI:** 10.3390/ijms251910264

**Published:** 2024-09-24

**Authors:** Hanjing Dai, Hao Ai, Yingrun Wang, Jia Shi, Lantian Ren, Jieqin Li, Yulu Tao, Zhaoshi Xu, Jiacheng Zheng

**Affiliations:** 1 College of Agronomy, Anhui Science and Technology University, Chuzhou 233100, China; 17605503449@163.com (H.D.);; 2 State Key Laboratory of Crop Gene Resources and Breeding, Institute of Crop Sciences, Chinese Academy of Agricultural Sciences (CAAS), Beijing 100081, China

**Keywords:** *Oryza sativa* L., *OsCCO* family, genome-wide analysis, phylogenetic association, collinearity relationship, abiotic stress response, tissue-specific expression

## Abstract

Carotenoid cleavage oxygenases (CCOs) cleave carotenoid molecules to produce bioactive products that influence the synthesis of hormones such as abscisic acid (ABA) and strigolactones (SL), which regulate plant growth, development, and stress adaptation. Here, to explore the molecular characteristics of all members of the OsCCO family in rice, fourteen *OsCCO* family genes were identified in the genome-wide study. The results revealed that the OsCCO family included one OsNCED and four OsCCD subfamilies. The OsCCO family was phylogenetically close to members of the maize ZmCCO family and the *Sorghum* SbCCO family. A collinearity relationship was observed between *OsNCED3* and *OsNCED5* in rice, as well as *OsCCD7* and *OsNCED5* between rice and *Arabidopsis*, *Sorghum,* and maize. OsCCD4a and OsCCD7 were the key members in the protein interaction network of the OsCCO family, which was involved in the catabolic processes of carotenoids and terpenoid compounds. miRNAs targeting *OsCCO* family members were mostly involved in the abiotic stress response, and RNA-seq data further confirmed the molecular properties of *OsCCO* family genes in response to abiotic stress and hormone induction. qRT-PCR analysis showed the differential expression patterns of *OsCCO* members across various rice organs. Notably, *OsCCD1* showed relatively high expression levels in all organs except for ripening seeds and endosperm. *OsNCED2a*, *OsNCED3*, *OsCCD1*, *OsCCD4a*, *OsCCD7*, *OsCCD8a*, and *OsCCD8e* were potentially involved in plant growth and differentiation. Meanwhile, *OsNCED2a*, *OsNCED2b*, *OsNCED5*, *OsCCD8b*, and *OsCCD8d* were associated with reproductive organ development, flowering, and seed formation. *OsNCED3*, *OsCCD4b*, *OsCCD4c*, *OsCCD8b*, and *OsCCD8c* were related to assimilate transport and seed maturation. These findings provide a theoretical basis for further functional analysis of the OsCCO family.

## 1. Introduction

Carotenoids are naturally occurring compounds classified as isoprenoid derivatives, predominantly appearing as orange or yellow pigments in many plants [1]. These compounds aid in the absorption and utilization of light energy and are potent antioxidants, preventing cellular damage caused by free radicals [2]. Additionally, carotenoids play a role in regulating plant growth and development and mitigate damage from various environmental stresses [3]. Carotenoid cleavage oxygenases (members of the CCO family) cleave carotenoid molecules to produce bioactive cleavage products such as retinal compounds, aroma compounds, and plant hormones; these products are essential for plant growth, development, and adaptation to stress [4]. The number of CCO family members varies across species. *Arabidopsis thaliana* has nine CCO family members, including *AtCCD1*, *AtCCD4*, *AtCCD7*, *AtCCD8*, *AtNCED2*, *AtNCED3*, *AtNCED5*, *AtNCED6*, and *AtNCED9* [5]; the human genome contains two CCO family members, *BCO1* and *BCO2*, which are primarily involved in carotenoid metabolism and maintaining vitamin A levels [6]. Various bacteria and fungi also have CCO family members with different numbers and biological types. These CCO family members share a conserved structure, i.e., the RPE65 domain, which binds iron ions and plays a crucial role in carotenoid metabolism [7].

Based on amino acid sequences and functional characteristics, the CCO family can be divided into two subfamilies as follows: the CCD subfamily (carotenoid cleavage dioxygenases) and the NCED subfamily (9-cis-epoxycarotenoid dioxygenases) [8]. The CCD subfamily regulates carotenoid metabolism, pigment synthesis, and hormone synthesis in plants. For example, tomato *LeCCD1* participates in the synthesis of essential flavor volatiles [9]. In plants such as honeysuckle and lily, *CCD4* is a key factor controlling the yellow or white coloration of floral organs [10]. In *Arabidopsis thaliana*, *AtCCD1* is upregulated under drought and salt stress, and the volatile products formed by cleaving carotenoids contribute to improving plant stress resistance [11]. *CCD7* and *CCD8* are involved in regulating the synthesis of the plant hormone strigolactone (SL), which controls processes such as plant aging, root growth, and tillering [12]. NCED catalyzes the cleavage of epoxycarotenoids and is the rate-limiting enzyme in abscisic acid (ABA) biosynthesis, playing a vital role in plant growth, development, and environmental adaptation [13]. Under drought conditions, maize *ZmNCED1* is significantly upregulated, leading to increased ABA levels, stomatal closure, reduced water loss, and enhanced drought resistance [14]. In rice, *OsNCED3* is highly expressed during seed maturation, accelerating ABA accumulation and promoting seed dormancy [15]. Under high salt conditions, tomato *SlNCED1* is upregulated, controlling stomatal closure and root growth to improve salt tolerance [16]. During cold stress, *BnNCED3* in rapeseed is upregulated, helping the plant adapt to low temperatures [17].

Rice (*Oryza sativa* L.) is one of the world’s most important food crops, providing sustenance for billions of people globally [18]. In 2002, the genome sequencing of the rice variety Nipponbare was completed in the International Rice Genome Sequencing Project, resulting in a highly accurate reference genome (T2T-NIP, AGIS-1.0 version) [19]. In recent years, rapid advances in rice genomics have significantly revealed the molecular mechanisms of rice stress resistance, plant architecture, and yield regulation, aiding in the development of rice varieties with superior agronomic traits [20]. The CCO family plays a crucial role in plant morphogenesis and ABA formation [21]. However, the *OsCCO* family genes in rice are relatively rarely reported, and the functions of most members during rice growth and development remain unclear. To further elucidate the structural characteristics and expression patterns of the *OsCCO* family members, this study conducted a genome-wide analysis to identify all members of the *OsCCO* family, and the phylogenetic associations, chromosomal locations, gene structures, and tissue-specific expression patterns of these family members were comprehensively examined to determine the molecular characteristics, providing a theoretical basis for further functional studies of the *OsCCO* family.

## 2. Results and Analysis

### 2.1. Family Identification, Physicochemical Properties, and Subcellular Localization Prediction

By scanning the whole rice genome (T2T-NIP/AGIS-1.0), 14 members of the *OsCCO* family were identified, including four *OsNCED* family genes and 10 *OsCCD* family genes. These candidate genes were named based on their homology genes in *Arabidopsis* (Table 1 and Table S1). The coding sequence lengths of the 14 genes of the *OsCCO* family ranged from 522 to 2406 bp, encoding proteins with 173 to 801 amino acids. The largest protein was OsCCD8b, and the smallest was OsNCED4a. The MW of these amino acids ranged from 18.65 to 97.93 kDa, with pI ranging from 4.77 to 9.19, and AI between 76.00 and 86.18. The INI values of OsNCED3, OsNCED2a, OsCCD8d, OsNCED5, and OsCCD1 were below 40, indicating their relatively stable proteins [22], whereas the others were predicted to be unstable. OsCCD8d and OsNCED2a had AI above 86, suggesting higher stability. GRAVY analysis showed that except for OsNCED2a, which displayed slight hydrophobicity, the rest of the OsCCO proteins were hydrophilic.

**Figure 1:**
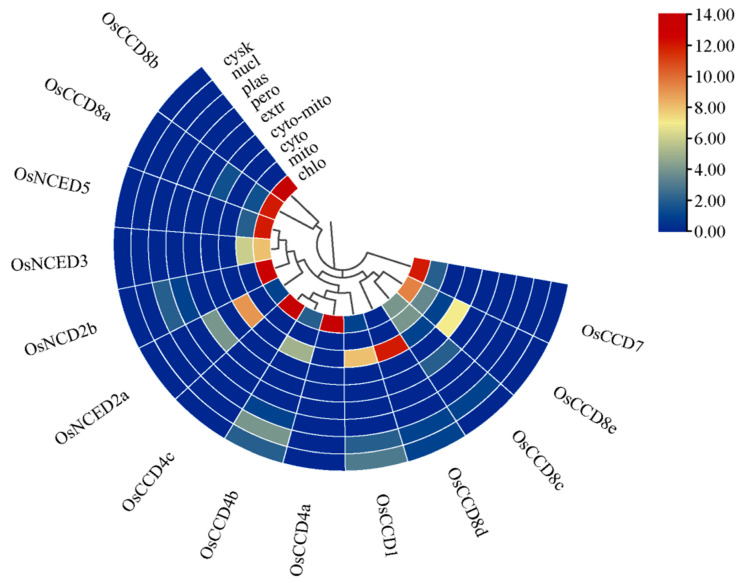
Subcellular localization of the OsCCO family members. Note: chlo is chloroplast, mito is mitochondria, cyto is cytoplasm, cyto-mito is cytoplasm or mitochondria, ext is extracellular matrix, pero is peroxisome, plas is plasma membrane, nul is nucleus, cysk is cytoskeleton. Detailed information on the OsCCO family members is listed in Table S1. The blue part of the bars indicates lower confidence in prediction results, whereas the red part indicates higher confidence.

The subcellular localization prediction indicated that most OsCCO proteins were distributed in the cytoplasm and chloroplasts, consistent with the finding that most OsCCO proteins are hydrophilic (Figure 1). The cytoplasm and chloroplasts are primarily involved in glycolysis, photosynthesis, hormone synthesis, and metabolism, suggesting that these OsCCO proteins may play roles in rice cell energy metabolism and growth processes [23].

### 2.2. Chromosomal Localization of the OsCCO Family

**Figure 2:**
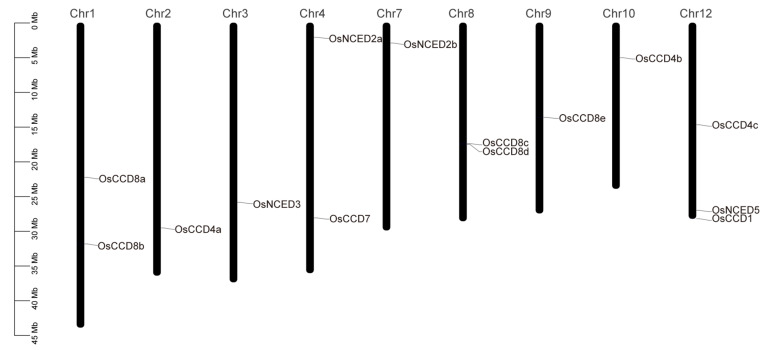
Distribution characteristics of the *OsCCO* family genes on rice chromosomes. Note: the chromosomal localization of the *OsCCO* family was created using TBtools v2.056 software. Detailed information on the *OsCCO* family genes is listed in Table S1.

The fourteen *OsCCO* family genes were located on nine chromosomes, with an uneven distribution (Figure 2). Chromosome 12 contained three *OsCCO* genes (*OsCCD4c*, *OsCCD1*, and *OsNCED5*), whereas chromosomes 2, 3, 7, 9, and 10 each contained only one *OsCCO* gene. *OsCCD8c* and *OsCCD8d* on chromosome 8 were located at the same position, suggesting the presence of gene clusters or gene duplication in this region, which may significantly affect gene expression regulation, functional specificity, and genetic evolution in rice. No *OsCCO* genes were found on chromosomes 5, 6, or 11. *OsCCD7* was located on chromosome 4; previous studies have shown that *OsCCD7* is involved in the synthesis of the plant hormone SL, which reduces plant height and increases tillering numbers of rice [24]. Future research will focus on utilizing *OsCCD7* for germplasm innovation during rice development.

### 2.3. Phylogenetic Analysis of the OsCCO Family Proteins

**Figure 3:**
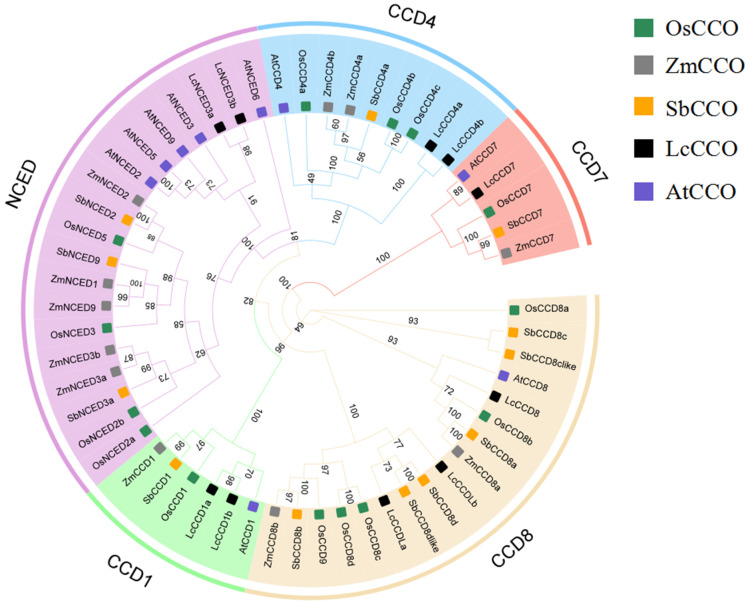
Phylogenetic association of plant CCO family proteins. Note: Os represents *Oryza sativa* L., Zm represents *Zea mays* L., Sb represents *Sorghum bicolor* L., Lc represents *Liriodendron chinense* (*Hemsl*.) Sarg, and At represents *Arabidopsis thaliana* L. The phylogenetic tree was constructed by way of neighbor-joining, with a bootstrap threshold of 1000. The numbers in the figure represent the phylogenetic distance between different CCO proteins. Detailed information on the CCO family members is listed in Table S2.

A phylogenetic tree was constructed using 56 CCO family proteins from five species including *Arabidopsis thaliana* L., *Liriodendron chinense* (*Hemsl*.) Sarg, *Oryza sativa* L., *Zea mays* L., and *Sorghum bicolor* L. The tree was grouped and named based on the *AtCCO* proteins in each branch (Figure 3). The results showed that the 56 CCO proteins were divided into the following five subfamilies: NCED, CCD4, CCD7, CCD8, and CCD1. Among them, the CCD8 subfamily was the largest, including five species, with rice members OsCCD8a, OsCCD8b, OsCCD8c, OsCCD8d, and OsCCD8e. The CCD7 subfamily was the smallest, containing only OsCCD7. Relative relationship comparison revealed that OsCCD4b and OsCCD4c were closely related to maize ZmCCD4a, ZmCCD4b, and *Sorghum* SbCCD4a. OsCCD7 was closely related to SbCCD7 and *ZmCCD7*. OsCCD8b was closely related to SbCCD8a and ZmCCD8a. OsCCD8a formed a separate branch, and OsCCD1 was also closely related to maize and *Sorghum*. In the NCED subfamily, four OsNCED proteins were closely related to *Sorghum* and maize NCED proteins and were distant from *Liriodendron* and *Arabidopsis* NCED proteins. These results indicate that the functions of rice CCO family members are similar to those in maize and *Sorghum*. CCD primarily participates in carotenoid cleavage, SL synthesis, and the regulation of plant growth and development, whereas the NCED is mainly involved in ABA biosynthesis, regulating seed germination and dormancy, stomatal closure, water management, and responding to drought, salinity, and disease stress [25].

### 2.4. Gene Structures, Functional Domains, and Conserved Motifs of the OsCCO Family 

**Figure 4:**
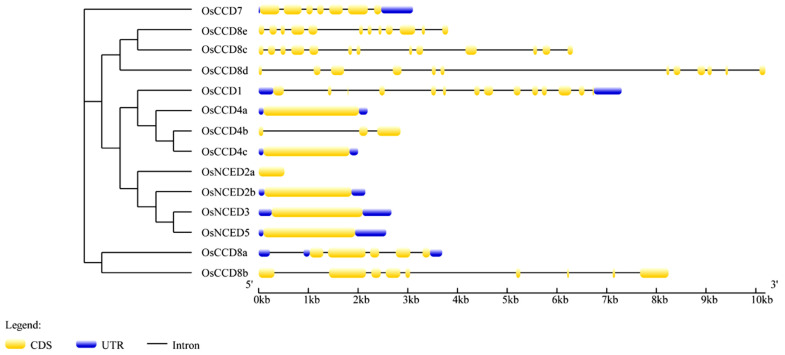
Structure analysis of *OsCCO* family genes. Note: The gene structures of the OsCCO family were created using the GSDS database (Table S3). Detailed information on the *OsCCO* family genes is listed in Table S1.

As shown in Figure 4, the number of exons in *OsCCO* family genes ranged from 1 to 13. The *OsCCD8* subfamily had a more complex distribution of introns and exons, with exon numbers ranging from 5 to 13. Among them, *OsCCD8c* had the most exons, i.e., 13 exons, and unlike other subfamilies, except for *OsCCD8a*, the rest of the *OsCCD8* subfamily genes lacked UTR regions. The gene structures of the *OsCCD4* and *OsNCED* subfamilies were simpler, with most genes containing only one exon, except for *OsCCD4b*. *OsCCD4a* had a long exon that was 1917 bp in length. *OsCCD4b* and *OsNCED2a* lacked UTR regions. Except for *OsNCED2a*, the single exons of the other *OsNCED* members were quite large, reaching over 1749 bp in length. UTR regions can regulate mRNA stability, translation efficiency, subcellular localization, and polyadenylation, finely modifying target gene expression. The 3′ UTR region of the *OsCCD7* gene was 636 bp, significantly different from the UTR-coding regions of the other *OsCCO* family members.

**Figure 5:**
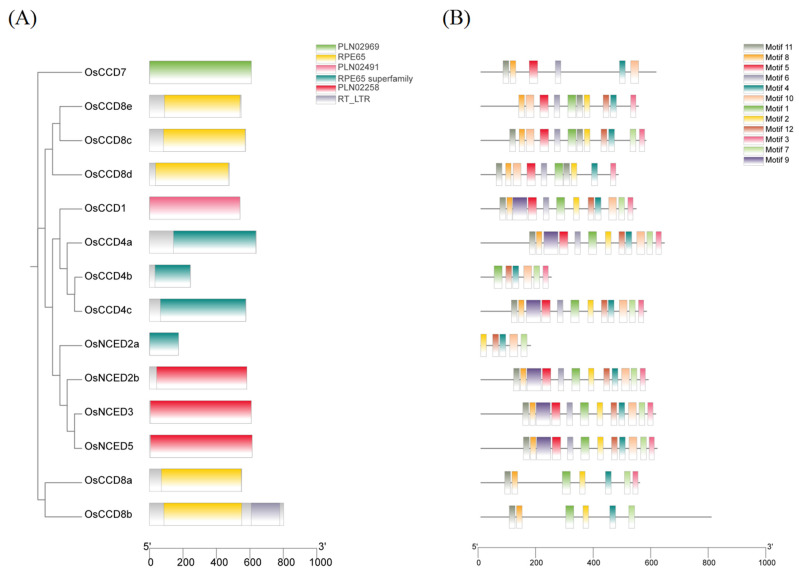
Functional domains and conserved motifs of OsCCO family proteins. (**A**) Conserved functional domains for the OsCCO family proteins. (**B**) Motif analysis for the OsCCO family proteins. Detailed information on the OsCCO family members is listed in Table S1.

The functional domain analysis revealed that the OsCCO family contained the following six types of domains: PLN02969, RPE65, PLN02491, RPE65-Superfamily, PLN02258, and RT-LTR. The PLN02969, PLN02491, and PLN02258 domains were members of the RPE65 family (Figure 5A). The results showed that OsCCD4a, OsNCED4b, OsCCD4c, and OsNCED2a possessed the RPE65 superfamily domain, whereas members of the OsCCD8 subfamily all contained the RPE65 domain, which is associated with carotenoid cleavage and SL and ABA synthesis [7]. Among them, OsCCD8b additionally included the RT_LTR domain, which may play a role in gene transposition and genome rearrangement [26]. OsNCED2b, OsNCED3, and OsNCED5 had the PLN02258 domain, which is primarily involved in ABA biosynthesis [27]. OsCCD7 and OsCCD1 contained the PLN02969 and PLN02491 domains, respectively. PLN02969 is related to SL biosynthesis and carotenoid cleavage [28], whereas PLN02491 is involved in carotenoid cleavage and pigment synthesis [29].

The conserved motif analysis showed that members with the RPE65 superfamily domain exhibited highly conserved motif structures (Figure 5B, Table S4, Supplementary Figure S1). OsCCD4a and OsCCD4c contained 12 identical motifs, whereas OsCCD4b and OsNCED2a were more conserved, sharing motifs 4, 7, 10, and 12. Members of the OsCCD8 subfamily, which contained the RPE65 domain, displayed highly conserved structures among OsCCD8c, OsCCD8d, and OsCCD8e. OsCCD8d lacked motifs 7 and 12, and OsCCD8e lacked motifs 7 and 11. Sequence alignment revealed 11 and 6 amino acid deletions in the motif 7 coding region of OsCCD8d and OsCCD8e, respectively. Additionally, OsCCD8a and OsCCD8b exhibited high conservation, both containing motifs 1, 2, 4, 7, 8, and 11. OsCCD8b lacked only motif 3, with OsCCD8b encoding 141 more amino acids in the motif 3 coding region compared with OsCCD8a. The members with the PLN02258 domain all contained 12 motifs, with sequence homology up to 76.03%. OsCCD7 and OsCCD1 contained 6 and 12 motifs, respectively, with OsCCD7 lacking motifs 1, 2, 3, 7, 9, and 12. These results indicate that proteins with the same conserved domains may have different motif distributions, potentially leading to functional divergence among members during evolution.

### 2.5. Cis-Acting Element Analysis of the OsCCO Family Gene Promoters

**Figure 6:**
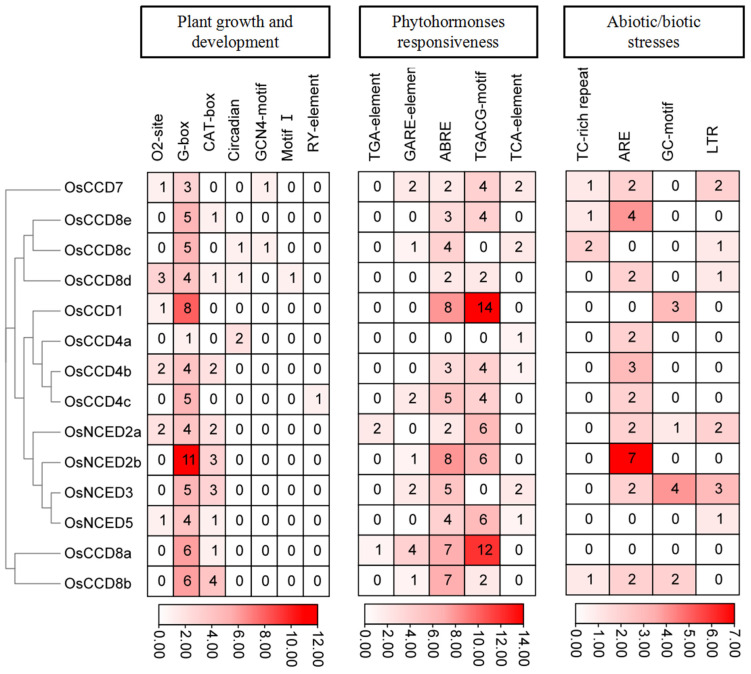
*Cis*-acting elements of promoters in *OsCCO* family genes. Note: the red part of the bar indicates more numbers of cis-acting elements in promoters, whereas the white part indicates fewer numbers. Detailed information on the *OsCCO* family genes is listed in Table S5.

Sixteen types of *cis*-acting elements of *OsCCO* family gene promoters were categorized into three groups (Figure 6 and Table S5). The first group comprised seven types with a total of 107 elements related to plant growth and development regulation, including maize prolamin response (O2-site), light response (G-box), meristem expression (CAT-box), circadian control (circadian), endosperm expression (GCN4-motif), root-specific regulation (motif I), and seed-specific regulation (RY-element). Light response elements accounted for the highest proportion of growth and development regulation elements at 66.36%, with all *OsCCO* family genes containing light signal response elements. The second group, related to plant hormones, included five types with a total of 149 elements, such as response to methyl jasmonate (MeJA, TGACG-motif), salicylic acid (SA, TCA-element), gibberellin (GA_3_, GARE-motif), abscisic acid (ABA, ABRE), and auxin (IAA, TGA-element). MeJA and ABA response elements accounted for 42.95% and 40.27%, respectively, with most *OsCCO* genes, except *OsCCD8c*, *OsCCD4a*, and *OsNCED3*, containing MeJA and ABA response elements. The third group, related to biotic and abiotic stress, included four types with a total of 53 elements, including defense and stress response (TC-rich element), anaerobic induction (ARE), anaerobic induction (GC-motif), and low temperature (LTR). Anaerobic induction response elements accounted for a relatively high proportion (52.83%), with most *OsCCO* genes, except *OsCCD8a*, *OsCCD1*, *OsCCD8c*, and *OsNCED5*, containing these elements. These findings suggest that the OsCCO family may play crucial roles in light-induced, hormone-regulated, and stress-resistance processes in plants.

### 2.6. Collinearity Analysis within the OsCCO Family and Homologous Evolutionary Associations among Different Species

**Figure 7:**
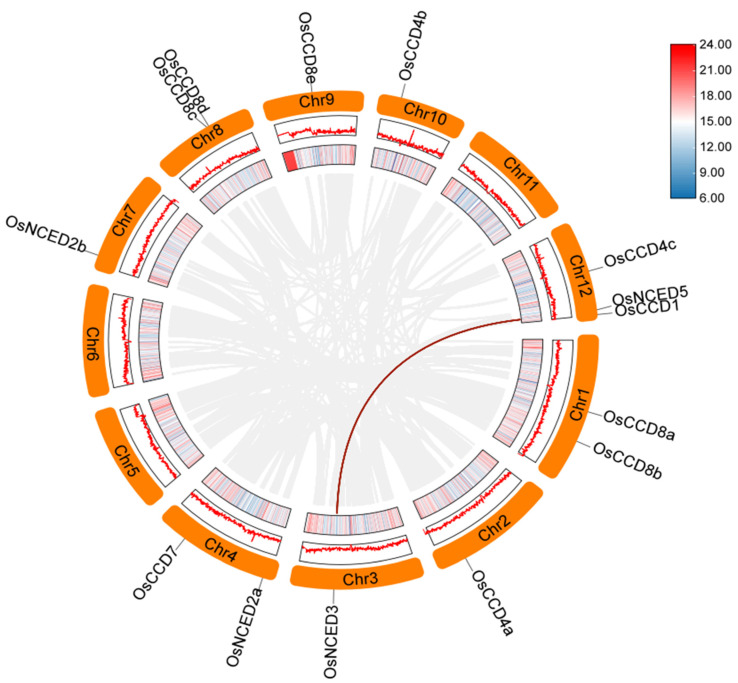
Collinearity analysis of the *OsCCO* family in the rice genome. Note: collinearity associations were analyzed by TBtools v2.056 software. Detailed information on the *OsCCO* family genes is listed in Table S1. The blue part of the bar indicates lower confidence in results, whereas the red part indicates higher confidence.

Within the rice genome, collinearity was found only between the *OsNCED3* gene on chromosome 3 and the *OsNCED5* gene on chromosome 12 (Figure 7, Table S6). The types of collinearity associations include whole-genome duplication (WGD) or segmental duplication on chromosomes. Since *OsNCED3* and *OsNCED5* are located on different chromosomes, this indicates a chromosomal segment duplication event during evolution.

**Figure 8:**
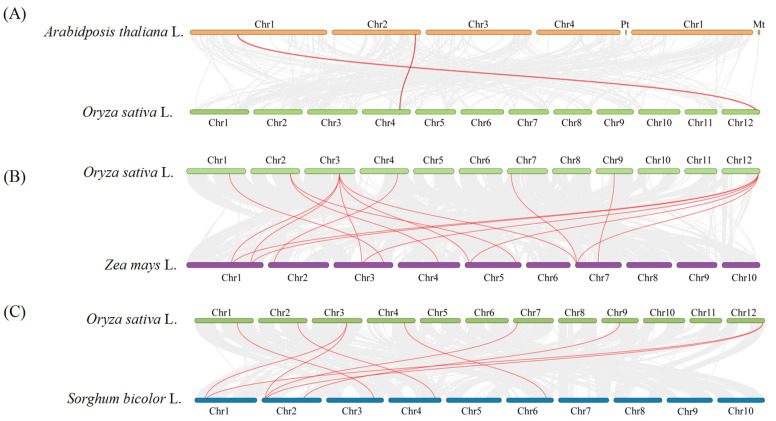
Collinearity analysis of the CCO family between rice and maize, *Sorghum,* and *Arabidopsis*. Note: (**A**–**C**) represented the collinearity associations between rice and *Arabidopsis,* maize and *Sorghum,* respectively. Collinearity associations were analyzed by TBtools v2.056 software. Detailed information on the *CCO* family genes in rice, *Arabidopsis*, *Sorghum*, and maize is listed in Table S6.

Through collinearity association analysis between the rice CCO family and *Arabidopsis*, maize, and *Sorghum,* the results revealed 2 collinear pairs between rice and *Arabidopsis*, 16 pairs between rice and maize, and 9 pairs between rice and *Sorghum* (Figure 8, Table S6). The collinear gene pairs have similar sequences because of their derivation from common ancestors, indicating a higher homology of the *OsCCO* family with the CCO family in maize and *Sorghum*, which is consistent with the evolutionary tree analysis. Genes with collinearity associations include *OsCCD8b*, *OsCCD4a*, *OsNCED3*, *OsCCD7*, *OsNCED2b*, *OsCCD8e*, and *OsNCED5*; the functions of these genes can be analyzed by referring to similar genes in *Arabidopsis*, maize, and *Sorghum*. Further analysis shows that *OsCCD7*, located on chromosome 4, and *OsNCED5*, located on chromosome 12, have collinearity associations in *Arabidopsis*, *Sorghum*, and maize, suggesting that both genes are highly conserved in position and function across different species.

### 2.7. Protein Interaction Analysis of the OsCCO Family

**Figure 9:**
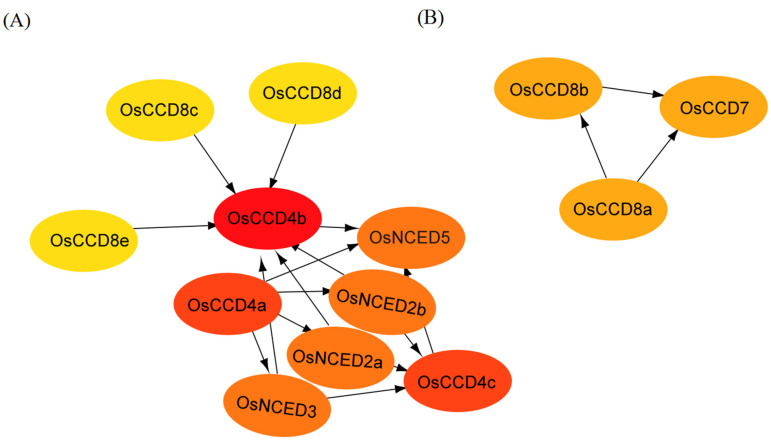
Protein interaction network of the OsCCO family. Note: (**A**,**B**) represent interaction networks centered on the OsCCD4b and OsCCD7 proteins, respectively. The protein interaction network of the OsCCO family was predicted using the STRING database, and the interaction network model was optimized and visualized using Cytoscape 3.9.1 software.

The OsCCO family proteins exhibit two independent interaction networks (Figure 9). The interaction network centered on the OsCCD4b is relatively complex, with OsCCD8c, OsCCD8d, and OsCCD8e upstream, regulating OsCCD4b and transmitting signals to OsNCED5. OsNCED3, OsNCED2a, and OsNCED2b simultaneously modify the effect of OsCCD4b and OsCCD4c. OsCCD4a regulates multiple members of the OsNCED family, with NCED being the rate-limiting enzyme for ABA biosynthesis, suggesting that OsCCD4a plays a significant role in ABA signal transduction. Additionally, OsCCD8a, OsCCD8b, and OsCCD7 form an independent interaction network, indicating that the protein interaction network centered on OsCCD7 is simple, with the metabolic process mainly influenced by OsCCD8a.

### 2.8. GO Annotation Analysis of the OsCCO Family

**Figure 10:**
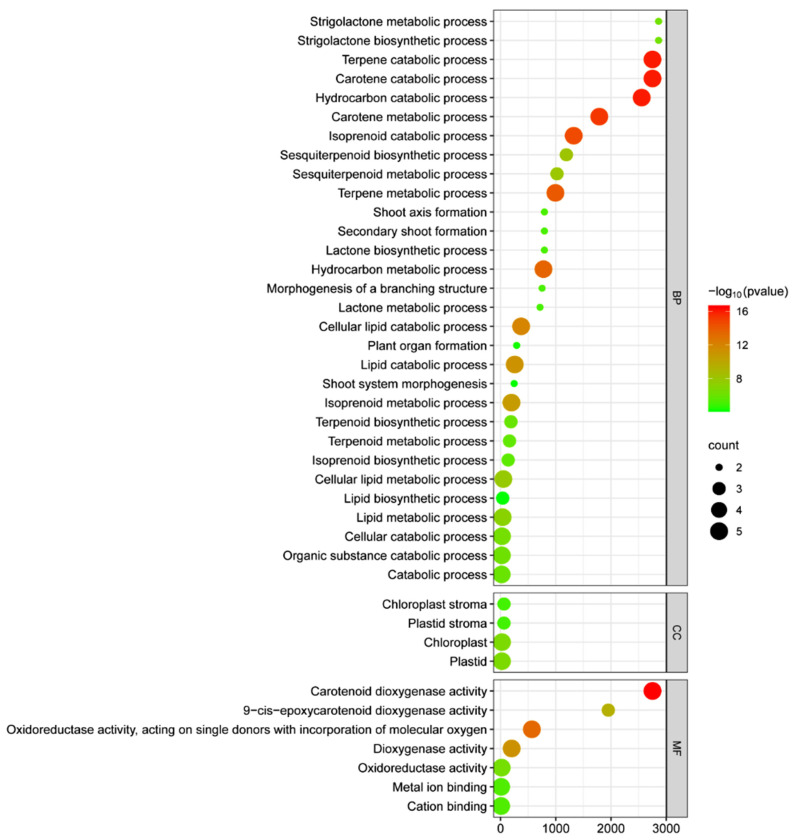
GO annotation analysis of the *OsCCO* family. Note: the molecular activity and biological processes of the OsCCO family members were analyzed using the ShinyGO database. The size of the dots represents the number of genes with corresponding GO annotations. The value on the horizontal axis indicates the degree of enrichment; the larger the value, the higher the enrichment. BP stands for biological process, MF for molecular function, and CC for cellular component.

Using GO enrichment analysis, the functions of the OsCCO family were elucidated from three aspects as follows: biological processes, molecular functions, and cellular components (Figure 10, Table S7). The results indicated that these genes are mainly involved in the carotenoid catabolic process (GO:0016121) and terpenoid catabolic process (GO:0046247) within the biological process category. In terms of molecular function, *OsCCO* genes are significantly associated with carotenoid dioxygenase activity (GO:0010436). Additionally, high enrichment of OsCCO proteins was found in the chloroplast stroma (GO:0009570). The comprehensive analysis suggests that the OsCCO family is closely related to carotenoid and terpenoid catabolic processes, highlighting their key roles in photosynthesis regulation, antioxidant defense, signal transduction, hormone regulation, secondary metabolite production, aroma and pigment formation, and the biosynthesis of signaling molecules.

### 2.9. Identification of miRNA Targeting OsCCO Family Genes in Genome-Wide of Rice

**Figure 11:**
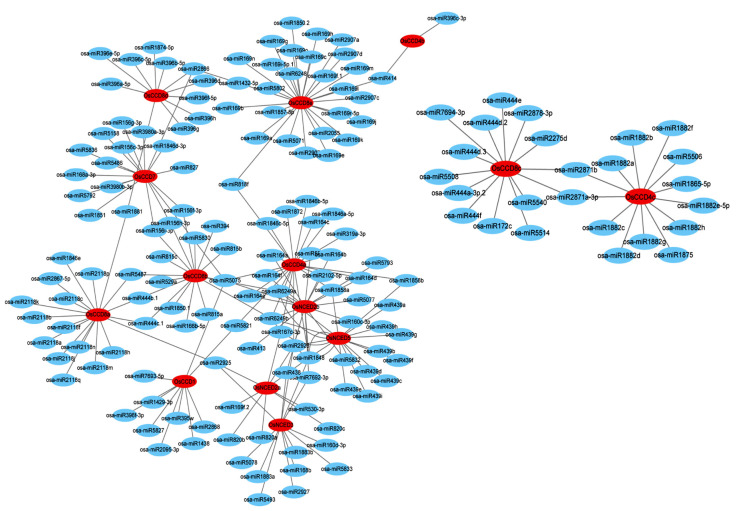
Interaction networks between *OsCCO* family genes and target miRNA molecules. Note: red ovals represent the 14 genes of *OsCCO* family, and blue ovals represent the miRNA molecules that target each gene of *OsCCO* family. The target miRNA molecules were predicted in the psRNATarget database, based on the sequences of *OsCCO* family genes. The interaction networks were mapped using Cytoscape 3.9.1 software.

A total of 164 miRNA molecules were identified as targeting 14 OsCCO family genes of rice (Figure 11, Table S8). The 164 miRNA molecules were divided into 81 groups, varying between 19 and 24 bp in length. Among them, Osa-miR5075, Osa-miR2926, and Osa-miR1848 targeted the most OsCCO family genes. Osa-miR5075 targeted OsCCD1, OsNCED2b, OsCCD7, OsCCD4a, and OsCCD8b. Osa-miR2926 targeted OsNCED5, OsNCED2b, OsNCED2a, OsNCED3, and OsCCD4a, and Osa-miR1848 targeted OsNCED5, OsNCED2b, OsNCED2a, OsNCED3, and OsCCD4a. The pathway analysis showed that some key miRNA molecules were associated with the environmental stress response, for example, the Osa-miR164, Osa-miR166, Osa-miR169, and Osa-miR1881 molecules were related to drought stress, which targeted the OsNCED2b, OsCCD4a, OsCCD1, OsCCD8b, OsCCD8e, OsNCED2a, OsCCD8d, and OsCCD8a genes, respectively. The Osa-miR156 and Osa-miR319 molecules were related to cold stress, which targeted the OsCCD7, OsCCD8b, and OsCCD4a genes. Osa-miR169g, Osa-miR169n, Osa-miR394, Osa-miR395, Osa-miR396c, and Osa-miR414 were associated with salt stress, which targeted the OsCCD8e, OsCCD8b, OsCCD1, OsCCD8d, and OsCCD4b genes. These results suggest that OsCCO family members of rice may be involved in abiotic stress responses in plants.

### 2.10. Expression Patterns in the OsCCO Family Genes in Different Rice Tissues

**Figure 12:**
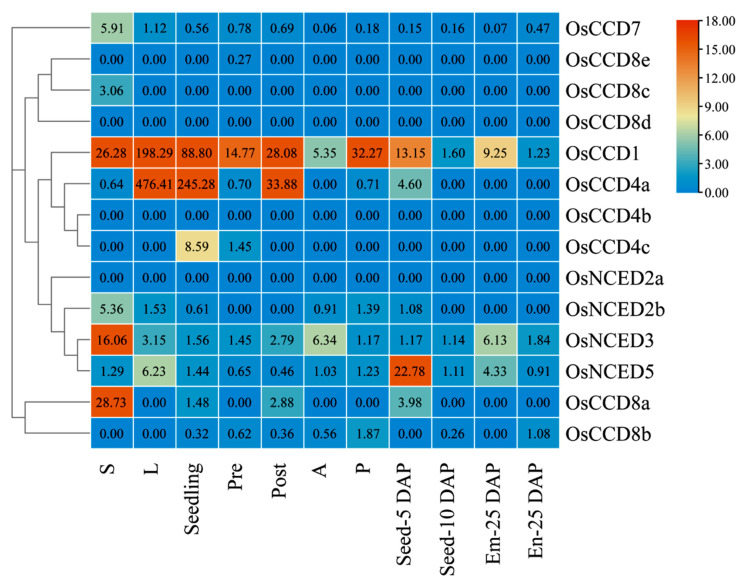
Tissue-specific expression patterns of *OsCCO* genes. Note: Ss is shoots, L is 20-day growth leaves, Seedling is four-leaf stage seedlings, Pre is pre-heading inflorescence, Post is post-heading inflorescence, A is anther, P is pistil, Seed-5 DAP is 5 d seeds after pollination, Seed-10 DAP is 10 d seeds after pollination, Em-25 DAP is 25 d embryos after pollination, En-25 DAP is 25 d endosperm after pollination. The red part of the bar indicates the highest expression level; the blue part of the bar indicates the lowest expression level.

As shown in Figure 12, the expression levels of the *OsCCO* family genes varied across different tissues. *OsCCD1* had relatively low expression levels in Seed-10 DAP and En-25 DAP but higher expression levels in other organs, with the highest expression in the L tissue. Within the *OsCCD4* subfamily, *OsCCD4a* exhibited higher expression levels in L, Post, and Seedling, whereas *OsCCD4c* showed relatively abundant expression only in Pre and Seedling. *OsCCD7* had low expression in A and Em-25 DAP but the highest expression in S. *OsCCD8a* showed higher expression in Ss and lower expression in Post and Seed-5 DAP. *OsCCD8b* was minimally expressed in P and En-25 DAP, whereas *OsCCD8c* was only slightly expressed in Ss. In the *OsNCED* subfamily, *OsNCED2b* and *OsNCED3* had higher expression levels in Ss, whereas *OsNCED5* showed higher expression in Seed-5 DAP. 

### 2.11. Expression Patterns of OsCCO Family Genes under Abiotic Stresses and Hormone Treatments

**Figure 13:**
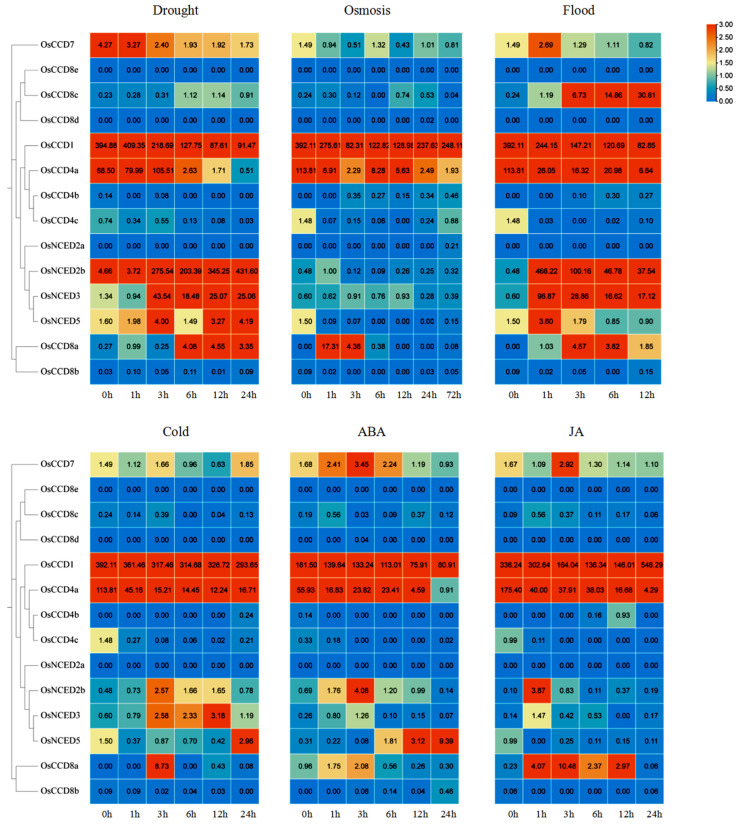
Expression patterns of *OsCCO* genes under abiotic stresses and hormone treatments. Note: drought is drought stress, osmosis is high-salt osmosis, flood is waterlogging, cold is cold injury, ABA is abscisic acid induction, and JA is jasmonic acid induction. The expression level was evaluated by RNA-seq data, which were downloaded from the NCBI database (Accession: PRJDB2600). Red part of the bar indicates the highest expression level; blue part of the bar indicates the lowest expression level.

The open RNA-seq data were used to identify the expression patterns of *OsCCO* family genes under abiotic stresses and hormone induction (Figure 13). The results showed that *OsCCD1* and *OsCCD4a* had relatively high expression levels at the seedling stage of rice, while *OsCCD8c* had relatively high expression levels under flooding stress. The expression levels of *OsNCED2b*, *OsNCED3*, *OsNCED5,* and *OsNCED8a* were significantly different among diverse treatments, and *OsCCD8d*, *OsCCD8e*, and *OsNCED2a* had almost no expression. For example, compared with the 0 h treatment (control), under drought stress, *OsCCD1* reached the highest expression level after 1 h, *OsCCD4a* and *OsNCED3* reached the highest expression level after 3 h, and *OsNCED2b* reached the highest expression at 24 h, which was 93 times that of control. The expression of *OsNCED8a* increased significantly after 6 h. 

Under high salt osmotic stress, *OsCCD1* and *OsCCD4a* had the highest expression levels in the control group, as well as *OsCCD8a* at 3 h. Under flooding stress, the expression levels of *OsCCD1* and *OsCCD4a* were highest in the control and decreased with the stress time extension. The expression levels of *OsNCED2b*, *OsNCED3*, and *OsNCED5* were the highest at 1 h, while *OsCCD8c* showed an increased expression after stress and reached the highest point at 12 h. After cold stress, *OsCCD1* and *OsCCD4a* showed a negative regulation, *OsNCED5* was higher at 24 h, and *OsNCED3* and *OsNCED2b* were relatively higher from 3 h to 12 h. Under ABA treatment, *OsCCD1* and *OsCCD4a* showed a downward trend. *OsCCD7*, *OsCCD8a*, and *OsNCED2b* showed the highest expression at 3 h, as well as *OsNCED5* at 24 h. For the jasmonic acid (JA) treatment, *OsCCD7* and *OsCCD8a* reached their maximum at 3 h, and *OsNCED2b* and *OsNCED3* accumulated an abundant expression level at 1h. These results suggest that the response characteristics of *OsCCO* family genes to different stresses in rice are quite different.

### 2.12. Expression Patterns of OsCCO Family Genes at Different Growth Stages

**Figure 14:**
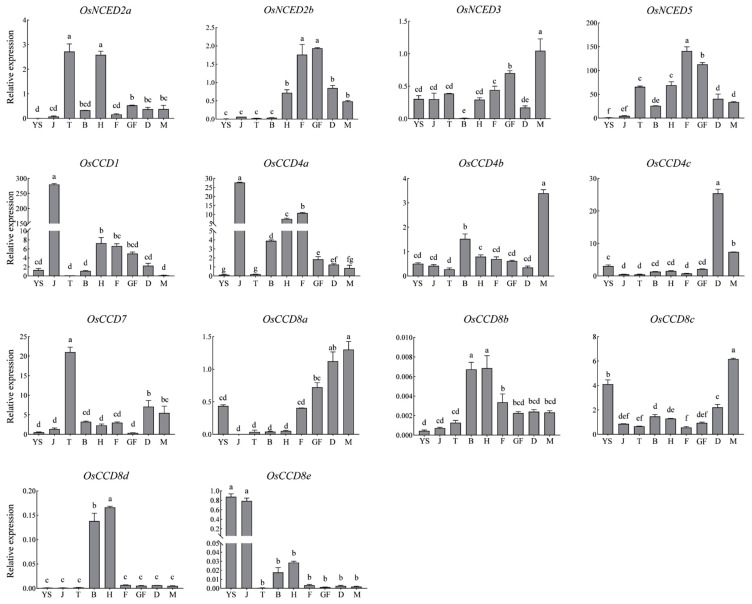
Expression patterns of *OsCCO* family genes in different growth stages. Note: YS is the young seedling stage, J is the jointing stage, T is the tillering stage, B is the booting stage, H is the heading stage, F is the flowering stage, GF is the grain-filling stage, D is the dough stage, M is the maturing stage. qRT-PCR data were analyzed using the 2^−ΔΔCt^ method to calculate relative expression levels of the *OsCCO* family genes. Different lowercase letters indicate significant differences (*p <* 0.05).

As shown in Figure 14, within the *OsNCED* subfamily, *OsNCED2a* had higher expression levels during the tillering and heading stages, *OsNCED2b* had significantly higher expression during the flowering and grain-filling stages, *OsNCED3* had the highest expression during the maturing stage, and *OsNCED5* showed higher expression during the flowering stage. The differential expression of these members suggests that the *OsNCED* subfamily has specific regulatory functions at different growth stages in rice, likely involved in the development of lateral buds, tillering, inflorescence formation, embryonic development during flowering, and grain filling and maturation [30]. In the *OsCCD4* subfamily, *OsCCD4a* and *OsCCD4b* showed higher expression levels during the jointing and maturing stages, respectively, whereas *OsCCD4c* was highly expressed during the dough stage. In the *OsCCD8* subfamily, *OsCCD8a* and *OsCCD8c* had higher expression levels during the maturing stage, whereas *OsCCD8b* and *OsCCD8d* showed higher expression during the booting and heading stages, indicating that *OsCCD8a* and *OsCCD8c* may play key roles in seed maturation and storage substance accumulation, whereas *OsCCD8b* and *OsCCD8d* have important functions in inflorescence development and young spike elongation. *OsCCD8e* was highly expressed only during the jointing stage. *OsCCD7* showed higher expression during the tillering stage, consistent with previous reports of its role in tiller formation and lateral bud development [31]. *OsCCD1* exhibited extremely high expression during the jointing stage, suggesting its critical function at this stage.

In summary, during the nine growth stages in rice, genes highly expressed during the seedling stage include *OsCCD8a*, *OsCCD8c*, and *OsCCD8e*; during the jointing stage, *OsCCD1*, *OsCCD4a*, and *OsCCD8e*; during the tillering stage, *OsNCED2a* and *OsCCD7*; during the heading stage, *OsNCED2a*, *OsCCD8b*, and *OsCCD8d*; during the flowering and grain-filling stages, *OsNCED2b* and *OsNCED5*; during the dough stage, *OsCCD4c*; and during the maturing stage, *OsNCED3*, *OsCCD4b*, *OsCCD8a*, and *OsCCD8c*. The differential expression of these genes at different growth stages reflects their specific regulatory functions in rice growth and development.

## 3. Discussion

The CCO family plays a crucial role in oxidizing and cleaving carotenoids to produce various important compounds, including plant hormones, pigments, aromas, and defensive substances. These compounds help protect against pathogens and herbivores, regulate plant adaptation to the environment, and influence tillering and growth [32]. Carotenoids are involved in the synthesis of hormones such as ABA and SL, as well as non-volatile compounds [33]. For example, CCD7 and CCD8 catalyze the formation of SLs, which inhibit lateral bud growth and promote root branching [34]. The SL signaling pathway can upregulate key *NCED* genes, promoting ABA synthesis at the stem base, whereas the ABA signaling pathway can inversely down-regulate *CCD7* and *CCD8*, inhibiting SL synthesis and thereby affecting plant branching and environmental resistance [35].

This study identified 14 *OsCCO* family genes in the rice genome (T2T-NIP/AGIS-1.0) through comparative analysis and predicted their biological functions and evolutionary associations. The results showed that most OsCCO proteins range from 91 to 600 amino acids in length, similar to the CCO family in other species, reflecting a certain level of evolutionary, structural, and functional conservation and diversity in CCO proteins [7]. Among the identified OsCCO proteins, OsNCED2a has slight hydrophobicity; the subcellular localization prediction of OsNCED2a in the cytoplasm suggests that its hydrophobicity may provide greater flexibility, allowing it to participate in processes related to plasma membrane formation or interactions with hydrophobic molecules [36]. The subcellular localization predictions showed that most OsCCO proteins are concentrated in the cytoplasm and chloroplasts, suggesting their functions may involve energy and material metabolism, such as photosynthesis, hormone regulation, and antioxidant defense [37]. The instability index indicates that the OsNCED3, OsNCED2a, OsCCD8d, OsNCED5, and OsCCD1 proteins are relatively stable, which could be advantageous for studies involving enzyme activity assays and protein–protein interactions [38]. 

Collinearity analysis revealed that *OsCCD7* and *OsNCED5* have collinearity associations in *Arabidopsis*, *Sorghum*, and maize, indicating they may be important target genes for crop improvement. Previous studies have shown that CCD7 plays a crucial role in SL biosynthesis and regulates branching patterns, thereby increasing crop yield [39]. *Arabidopsis* AtNCED5 is a key factor regulating ABA synthesis under drought stress [30], indicating significant research and application value for OsCCD7 and OsNCED5. In fact, a few transgenic plants with *OsCCD7* gene knockout had been obtained by agrobacterium-mediated transformation. To increase tillering numbers greatly and reduce plant height in T_0_ offspring, the detailed information needs to be further investigated.

Exons and introns play important roles in gene evolution, with the number and location of introns influencing gene expression levels and regulatory mechanisms [40]. This study shows that members within the same subfamily have similar exon and intron distributions, with the *OsNCED* subfamily showing higher structural conservation compared to the *OsCCD* subfamily, suggesting less gene variation or loss during evolution. Different functional domains endow proteins with specific biosynthetic capabilities, affecting plant morphology, color, and environmental responses [41]. The OsCCO family contains various conserved functional domains, and the results revealed that factors with the same functional domains also have different motif numbers. For example, OsCCD4a, OsCCD4b, OsCCD4c, and OsNCED2a all have the RPE65 superfamily domain, but OsCCD4a and OsCCD4c are highly conserved in motif structure, whereas they differ significantly in OsCCD4b and OsNCED2a, suggesting differences in functions related to carotenoid cleavage, ABA biosynthesis, and substrate specificity [7]. Within the RPE65 domain, except for OsCCD8c, which contains 12 motifs, the other members show varying degrees of motif loss. Members with the PLN02258 domain, OsNCED2b, OsNCED3, and OsNCED5, are highly conserved, indicating similar biological functions in environmental stress responses and ABA synthesis [27]. These findings provide clues to understanding the regulatory mechanisms of the rice OsCCO family in various biological metabolic processes.

*Cis*-regulatory elements in promoters are crucial for regulating gene transcription levels, with a rich array of response elements reflecting the necessity for plants to control physiological processes precisely through complex signaling pathways [4]. The promoter cis-elements of the *OsCCO* family genes can be categorized into three groups as follows: plant growth and development, plant hormone synthesis, and biotic/abiotic stress responses. The high proportion of light-responsive elements related to plant growth and development suggests that the *OsCCO* family enables plants to perceive and respond to changes in environmental light effectively, optimizing growth and development and enhancing competitive survival and adaptability [42]. Additionally, the *OsCCO* family contains numerous anaerobic response elements, likely because rice frequently faces flooding and hypoxic conditions.

The GO enrichment analysis revealed that OsCCO is mainly involved in the catabolic processes of carotenoids and terpenoids. Carotenoids protect chloroplasts from light damage during photosynthesis and are involved in light capture and energy transfer [43]. Furthermore, carotenoids and their degradation products play key roles in the antioxidant defense system of plants, helping plants resist oxidative stress [44]. Terpenoid compounds regulate plant growth and development, attract pollinators, repel herbivores, and protect plants from pathogens and insects [45]. Thus, OsCCO is an important factor in regulating plant growth and development and responding to environmental stress, providing valuable candidate genes for crop improvement and enhancing plant stress resistance.

In rice, miR164 targets the *OMTN2*, *OMTN3*, *OMTN4*, and *OMTN6* genes and upregulates many key factors related to stress, development, and metabolism. *OsHB4* targeted by miR166 is also an important regulator in plant development [46]. The main target of miR166 is the *OsHB4* gene, and rice plants with miR166 knockout show leaf curl, reduced transpiration, and enhanced drought tolerance [47]. miR169g and miR1881 are related to drought stress. Under drought treatment, miR169g expression is down-regulated and more sensitive in rice roots, while miR1881 expression shows great variance in different varieties [48,49]. miR156, targeting the *OsSPL3* gene, negatively regulates the transcription factors OsMYB2 and OsMYB3R-2 to improve the cold resistance of rice [50]. Under salt stress, the expression level of miR414 is down-regulated, which improves the salt resistance of rice [51], and miR396c is also down-regulated under NaHCO_3_ alkaline treatment [52]. Based on the expression patterns of *OsCCO* family genes under stress, *OsNCED2b*, *OsCCD4a*, and *OsCCD1*, all targeted by Osa-miR164, and *OsCCD8a*, targeted by Osa-miR1881, are estimated to be involved in drought stress. *OsCCD7* and *OsCCD8b*, targeted by Osa-miR156, and *OsCCD4a*, targeted by Osa-miR319, are related to cold stress. *OsCCD1*, targeted by Osa-miR396f, and *OsCCD4b* targeted by Osa-miR396c, are associated with salt stress.

The differential expression of target genes in different tissues or organs reflects the stage-specific regulation of target genes in plant growth, development, tissue differentiation, and environmental adaptation. *AtNCED3* in *Arabidopsis* is highly expressed in root tip meristems [53], *ZmCCD7* in maize is expressed in roots, stems, leaves, and spikes, with especially highest expression in roots [40], and *CpCCD8* in meratia praecox is mainly expressed in roots and axillary buds [54]. In this study, *OsCCD7*, *OsCCD8c*, *OsCCD8a*, *OsNCED2b*, and *OsNCED3* showed higher expression in shoots, indicating that these genes may play key roles in the early stages of rice growth, which are partly consistent with previous studies. *VvCCD1* and *VvCCD4* in grapes are mainly expressed in leaves [55]; here, *OsCCD1* and *OsCCD4a* exhibited the highest expression in leaves, with the *OsCCD1* promoter containing multiple light-responsive elements, as well as the *OsCCD4a* promoter related to circadian regulation and anaerobic induction, suggesting these factors may participate in photosynthesis and energy conversion metabolism in leaves. *OsNCED5* showed higher expression in seeds, indicating its role in seed development and material accumulation.

The *CaNCED2* and *CaCCD5* genes of chickpeas are positively correlated with an improvement in drought resistance, as well as the *LcNCED2*, *LcCCD1*, and *LcCCD2* genes in lentil genes [56]. Here, *OsCCD4a* and *OsNCED2b* were also induced by drought stress. In soybeans, the transcription levels of *NCED* family genes increased after ABA treatment, and *CCD7* and *CCD8* significantly increased after a 6 h treatment, indicating that *CCO* family members are closely related to the ABA-mediated regulatory network [57]. In this study, the transcription levels of *OsCCD7* and *OsCCD8a* significantly increased after a 3h treatment of ABA, which was consistent with previous studies. The *NCED* family members of *Arabidopsis* are a key rate-limiting enzyme in the ABA synthesis pathway and participate in ABA biosynthesis, and ABA is very important for plant development and the stress response [58]. Here, it was found that *OsNCED2b*, *OsNCED3*, and *OsNCED5* of the *NCED* subfamily were all induced to increase the transcription levels under ABA treatment, which also confirmed the important role of plant *NCED* genes in the ABA signaling pathway.

In different growth stages, *OsNCED2a*, *OsCCD1*, *OsCCD4a*, *OsCCD7*, and *OsCCD8e* exhibited higher expression in the early development stages (seedling, jointing, and tillering stages), suggesting these genes are involved in early plant growth and differentiation. *OsNCED2a*, *OsNCED2b*, *OsNCED5*, *OsCCD8b*, and *OsCCD8d* showed significantly higher expression during the mid-growth stages (booting, heading, and flowering stages), indicating their crucial roles in reproductive growth processes such as flowering and fruiting. *OsNCED3*, *OsCCD4b*, *OsCCD4c*, *OsCCD8b*, and *OsCCD8c* showed increased expression during the late growth stages (grain-filling, dough, and maturing stages), indicating their importance in maturation and grain filling, likely related to assimilating transport and seed maturation.

## 4. Materials and Methods

### 4.1. Experimental Materials

The rice variety Nipponbare was used, potted at Anhui Science and Technology University in May 2023. Leaves without pests or damage were collected during the following growth stages: seedling, tillering, jointing, booting, heading, flowering, filling, dough, and maturity. Each sample had three replicates, which were quickly frozen in liquid nitrogen and ground. Total RNA was extracted using the Trizol reagent (T9108, Takara Japan) and reverse-transcribed into a cDNA sample using a KIT (Vazyme, R323-01, Nanjing, China).

### 4.2. Characteristics of the OsCCO Family of Rice

#### 4.2.1. Identification of the OsCCO Family

Nine reported CCO family sequences were searched in the TAIR database of *Arabidopsis* and compared with the rice whole genome database, identifying 14 candidate *OsCCO* sequences. Protein structures were predicted using the online tool CDD of NCBI (National Center for Biotechnology Information), and proteins lacking the conserved RPE65 domain were removed. The identified protein sequences were validated using the PfamScan program, yielding candidate *OsCCO* family members. Gene names were assigned based on homologous genes in *Arabidopsis*, and genes within the same subfamily were sequentially named by lowercase letters according to their chromosomal locations. The rice whole-genome data, protein sequences, and annotation files were downloaded from the RiceSuperPIRdb database. Database information is listed in Table S3.

#### 4.2.2. Physicochemical Properties and Subcellular Localization of the OsCCO Family

The ExPASy database was used to determine the amino acid length, molecular weight (MW), isoelectric point (pI), instability index (INI), aliphatic index (AI), and grand average of hydropathy (GRAVY) of the OsCCO family. The subcellular localization characteristics of OsCCO family members were predicted using the WoLF PSORT database.

#### 4.2.3. Chromosomal Localization and Phylogenetic Tree Construction of the OsCCO Family

A chromosomal localization map of the OsCCO family was created using TBtools v2.056 software [59]. Fifty-six protein sequences of the CCO family from five species were selected to construct a phylogenetic tree using MEGA 11.0 software (neighbor-joining, bootstrap threshold with 1000) and visualized using the Evolview online tool. The proteins in the CCO family are listed in Table S2.

#### 4.2.4. Conserved Motifs, Functional Domains, Gene Structures, and Promoters of the OsCCO Family

The online MEME tool was used to analyze the conserved motifs and functional domains of the CCO family proteins [60]. The gene structure map of the *OsCCO* family was created using the GSDS database [61]. The types, numbers, and functions of *cis*-elements in their promoters were predicted using the PlantCARE database [62].

#### 4.2.5. Collinearity Analysis of the OsCCO Family Genes

The published genome data and annotation information of *Arabidopsis*, rice, maize, and *Sorghum* were downloaded, and collinearity associations within the rice *OsCCO* family, and between rice and other species, were analyzed by TBtools v2.056 software. The Advanced Circos in TBtools v2.056 software was used to perform the visualization drawing. Collinearity data are listed in Table S6.

#### 4.2.6. Protein Interaction and GO Enrichment Analysis of the OsCCO Family

The protein interaction network of the OsCCO family was predicted using the STRING database, and the interaction network model was optimized and visualized using Cytoscape 3.9.1 software [63]. The molecular activity and biological processes of the OsCCO family members were analyzed using the ShinyGO database [64], with a false discovery rate (FDR) at *p* < 0.01 for GO enrichment analysis [65].

#### 4.2.7. Genome-Wide Analysis of miRNA Targeting OsCCO Family Genes

Based on the sequences of *OsCCO* family genes of rice, the target sites of target genes were predicted in the psRNATarget database, to search related miRNA molecules, and the interaction networks between target genes of *OsCCO* family and target miRNA molecules were mapped using Cytoscape 3.9.1 software [63].

### 4.3. Tissue-Specific Expression of the OsCCO Family Members

Transcriptome data, including their expression levels (FPKM values) in shoots, leaves (20 d old), young seedlings (four-leaf stage), inflorescences (pre- and post-heading), anthers, pistils, seeds (5 d and 10 d post-fertilization), young embryos (25 d post-fertilization), and endosperm (25 d post-fertilization), were downloaded from the RGAP database of rice. Data were transformed with the formula log_2_(FPKM + 1) to calculate gene expression levels and visualized using TBtools v2.056 software. The expression levels of the *OsCCO* family members at different growth stages were detected using real-time quantitative PCR (qRT-PCR) with cDNA from rice leaves as templates. qRT-PCR procedures and reaction systems followed Zheng’s protocol [66]. The primers are listed in Table S9.

### 4.4. Expression Patterns of OsCCO Family Genes under Abiotic Stress and Hormone Treatments

The 14d sprouted rice seedlings (*Oryza sativa* Japonica Group) were used as target materials to download the RNA-seq data under various environmental conditions in the NCBI database (Accession: PRJDB2600) to evaluate the expression patterns of *OsCCO* family genes under drought, high salt penetration, waterlogging, cold injury, abscisic acid, and jasmonic acid. By using the SRAtoFastq plug-in of TBtools v2.056 software to control and filter original reads, and using the Kallisto Super Wrapper to map the reference genome, standardized TPM (Transcripts Per Kilobase Million) values are generated. The HeatMap plug-in of TBtools was used to create a heat map for the expression patterns of *OsCCO* family genes.

### 4.5. Data Processing

qRT-PCR data were analyzed using the 2^−ΔΔCt^ method to calculate relative expression levels [67]. Expression levels were statistically analyzed using one-way ANOVA in SPSS18.0 software, with significance determined by Duncan’s multiple comparison test *(p <* 0.05). Data were organized and plotted using GraphPad Prism 8.0 software.

## 5. Conclusions

Fourteen OsCCO family members were identified in the rice genome, including four *OsNCED* genes and ten *OsCCD* genes. Subcellular localization predictions indicated that OsCCO proteins were mostly concentrated in the cytoplasm and chloroplasts, with most proteins being unstable in vitro. The OsCCO family in rice is closely related to the maize ZmCCO family and the *Sorghum* SbCCO family. *OsCCD7* and *OsNCED5* exhibited collinearity associations between rice and *Arabidopsis*, *Sorghum*, and maize. OsCCD4a and OsCCD7 are key members of the OsCCO family protein interaction network, mainly involved in the catabolic processes of carotenoids and terpenoid compounds. The promoters of *OsCCO* family genes contain many *cis*-elements related to light response, MeJA, ABA, and other factors. miRNA molecules targeting *OsCCO* family genes were mostly involved in abiotic stress responses in rice, such as drought, cold injury, high salt stress, etc. RNA-seq data further confirmed similar functional properties, and qRT-PCR suggested that *OsNCED2a*, *OsNCED3*, *OsCCD1*, *OsCCD4a*, *OsCCD7*, *OsCCD8a*, and *OsCCD8e* were related to plant growth and differentiation, whereas *OsNCED2a*, *OsNCED2b*, *OsNCED5*, *OsCCD8b*, and *OsCCD8d* were associated with reproductive organ development, flowering, and seed formation. *OsNCED3*, *OsCCD4b*, *OsCCD4c*, *OsCCD8b*, and *OsCCD8c* were related to assimilate transport and seed maturation. These results lay the foundation for further functional analysis of the OsCCO family.

## Figures and Tables

**Table 1:** Physicochemical properties of the OsCCO family members.

Gene Name	Gene ID	CDS Length (bp)	Amino Acids (aa)	MW (kDa)	pI	INI	AI	GRAVY
*OsCCD1*	LOC_Os12g44310	1623	540	60.95	5.9	32.57	76.44	−0.322
*OsCCD4a*	LOC_Os02g47510	1917	638	68.62	6.07	41.95	77.04	−0.148
*OsCCD4b*	LOC_Os10g08980	738	245	27.18	4.77	49.26	76.00	−0.171
*OsCCD4c*	LOC_Os12g24800	1731	576	63.87	6.26	42.17	85.62	−0.067
*OsCCD7*	LOC_Os04g46470	1830	609	68.19	9.19	51.65	77.55	−0.295
*OsCCD8a*	LOC_Os01g38580	1659	552	59.92	5.88	50.25	78.13	−0.188
*OsCCD8b*	LOC_Os01g54270	2406	801	87.93	5.89	46.69	80.24	−0.259
*OsCCD8c*	LOC_Os08g28240	1725	574	64.63	6.33	46.29	82.33	−0.268
*OsCCD8d*	LOC_Os08g28410	1437	478	53.64	6.04	35.09	86.63	−0.213
*OsCCD8e*	LOC_Os09g15240	1647	548	61.07	5.84	40.88	85.24	−0.181
*OsNCED2a*	LOC_Os04g04230	522	173	18.65	5.37	36.21	86.18	0.029
*OsNCED2b*	LOC_Os07g05940	1749	582	62.95	6.21	46.06	82.30	−0.233
*OsNCED3*	LOC_Os03g44380	1827	608	65.65	5.77	36.74	77.57	−0.172
*OsNCED5*	LOC_Os12g42280	1842	613	65.93	5.83	38.62	76.61	−0.140

Note: MW is molecular weight, pI is isoelectric point, INI is instability index, AI is aliphatic index, and GRAVY is the grand average of hydropathy.

## Data Availability

The data in this study will be shared by the corresponding authors if reasonably requested for study.

## Supplementary Materials

The following supporting information can be downloaded at: https://www.mdpi.com/article/10.3390/ijms251910264/s1.
