# Supplementary material for: Molecular Characteristics and Expression Patterns of Carotenoid Cleavage Oxygenase Family Genes in Rice (Oryza sativa L.)
Source: Int J Mol Sci. 2024 Sep 24;25(19):10264. doi: 10.3390/ijms251910264 (PMC11477027; doi:10.3390/ijms251910264)
Supplement: Supplementary file 1 [file ijms-25-10264-s001.zip › Supplementary Figure S1.pdf]

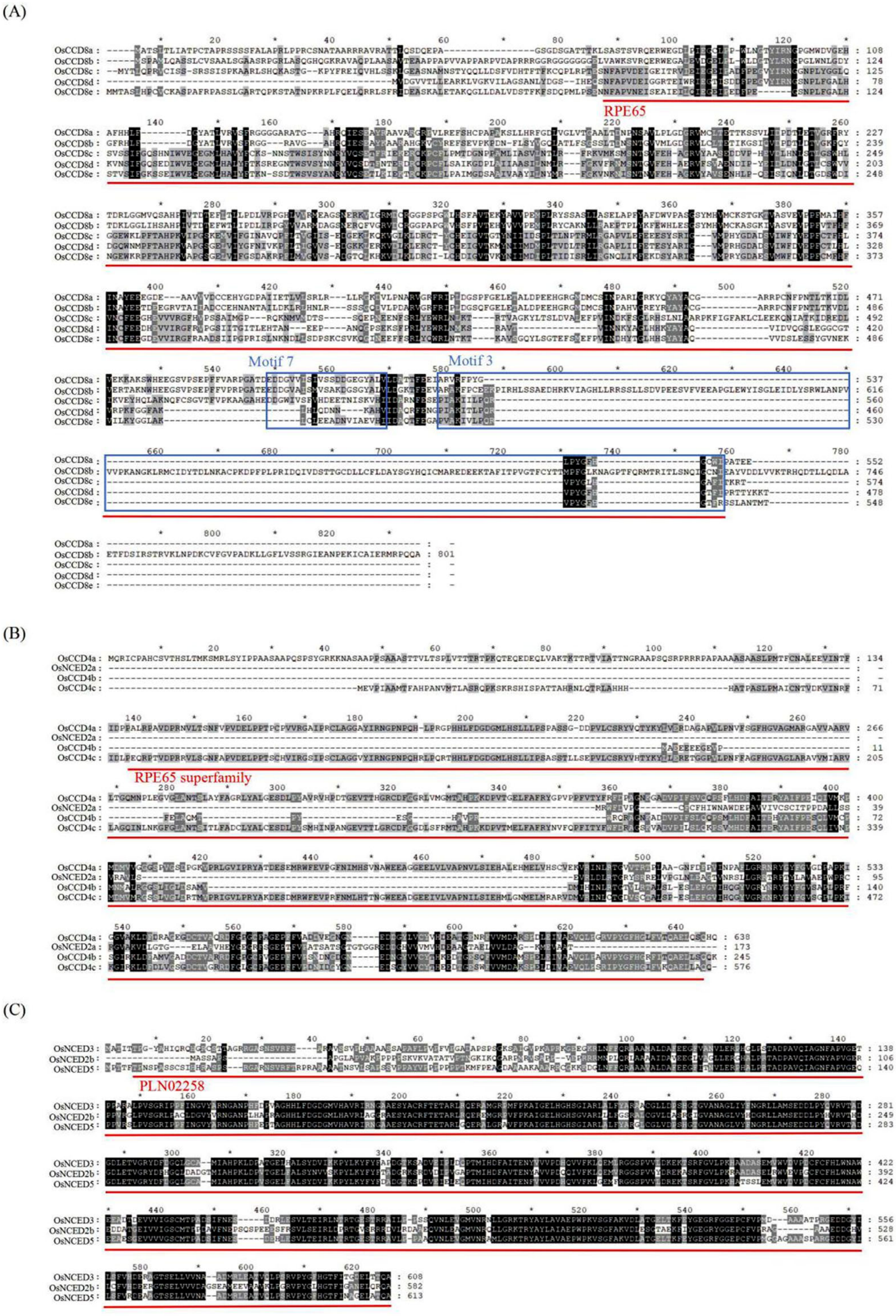

Supplementary Figure S1. Conserved functional domains of the OsCCO family proteins. Note: the OsCCO family contains six types of functional domains: PLN02969, RPE65, PLN02491, RPE65-Superfamily, PLN02258, and RT-LTR. (A), (B), and (C) represent the RPE65, RPE65 superfamily, and PLN02558 conservative domains, respectively, and the PLN02969, PLN02491, and RT-LTR conservative domains all contain only one member. The red underline indicates the location of the conservative functional domain, and the blue wireframe indicates the key motif 3 and motif 7 structural scope.
